# Nurturing empowered scholars: A student perspective on contemporary feminist mentorship

**DOI:** 10.1177/09593535251327863

**Published:** 2026-01-27

**Authors:** Storm Balint, Gena K. Dufour, Jewels Adair, Ngai Lam Mou

**Affiliations:** 8637 University of Windsor, Canada; 8637 University of Windsor, Canada; 8637 University of Windsor, Canada; 8637 University of Windsor, Canada

**Keywords:** empowerment, feminist mentorship, graduate student success, inclusivity, intersectionality

## Abstract

In this collaborative commentary, four PhD students in a feminist psychology research lab present a student-focused perspective on the importance of feminist mentorship within academia. We define feminist mentorship as inclusive, intersectional, and responsive to our differing backgrounds and experiences. Feminist mentoring aims to deconstruct traditional understandings of power in academic spaces. Feminist mentorship has profoundly shaped our educational journeys and feminist identities, highlighting the crucial role of diverse mentorship from the start of a student's academic career. Essential for graduate student success in competitive and often isolating academic environments, feminist mentors support emerging feminists, advocate students’ interests, and empower them to advocate for themselves. Having laid the foundation for inclusive and dynamic feminist mentorship, we look ahead to future challenges and opportunities for students and mentors. Drawing from our experiences, we offer recommendations on how to best support emerging feminist scholars. This involves connecting with other feminists for networking and collaboration, actively seeking funding opportunities, and providing support and guidance for community-based advocacy. Our collective perspective highlights the essential role of feminist mentoring. We advocate a continued focus on enhancing and prioritizing feminist mentorship in academia and acknowledging its significant influence on the growth of empowered scholars.

Mentorship in academia often involves power dynamics where one party holds more authority (Cobb et al., 2006). Graduate students have less power and status when interacting with professors or university administrators due to their lack of authority. In addition, factors such as gender, race/ethnicity, and socioeconomic status could also contribute to the varying power levels between graduate students and their mentors. Understanding these varying power and influence levels within the academic hierarchy is crucial, as they shape graduate students’ perspective on mentorship.

Awareness of the impact of power on graduate student mentees gives rise to an alternative approach: feminist mentorship, which provides a nuanced way to navigate the complexities of mentorship within academia (Benishek et al., 2004; Manongsong & Collins, 2022). We conceptualize feminism as a positive value that challenges systemic inequities and cultural assumptions about social hierarchy. Feminist principles foster inclusive environments, critical reflexivity, community, solidarity, empowerment, and more. Unlike traditional models, feminist mentorship challenges conventional power dynamics and fosters inclusive environments (Benishek et al., 2004). Feminist mentorship offers graduate students, particularly women, a supportive framework to navigate academia's complexities by advocating equity, diversity, and inclusivity. This transformative shift in mentorship reshapes students’ understanding of traditional dynamics and promotes a more empowering academic experience.

In the following sections, we explore the impact of feminist mentorship on our academic journeys, particularly graduate education. As four PhD students from a feminist psychology research lab at a university in Southern Ontario, Canada (Table 1), we have diverse experiences with feminist mentorship but aim to highlight how it has been pivotal in shaping our growth as feminist scholars.

**Table 1.** Researcher descriptions detailing professional roles and experiences.

**Jewels Adair** is a PhD candidate focusing on the study of gaslighting and other manipulative tactics used by perpetrators of gender-based violence, along with the social-psychological outcomes experienced by survivors. She is a White, heterosexual woman from a working-class background and the first in her family to pursue a university degree.
**Storm Balint** is a PhD candidate researching women's diverse experiences with sexual agency and its impacts stemming from encounters with sexual violence, specifically sexual coercion. She is a White, heterosexual, cisgender woman raised in a working-class family.
**Gena K. Dufour** is a PhD candidate specializing in applied forensic and social psychology. She is a fat, queer, cisgender woman who is half Métis (nontreaty status) and half White. Gena comes from a working-class background and is a first-generation academic. She studies the way victims of gender-based violence interact with larger systems and institutions in Canada, including survivor perceptions of procedural justice and institutional betrayal.
**Ngai Lam Mou** is a PhD candidate from Macau studying in Canada as an international student. She investigates men's perpetration of sexual coercion against women, with a broad research interest in the prevention of sexual violence. Coming from a working-class family, Ngai Lam is also a first-generation academic.

Our experiences with feminist mentorship extend beyond academia. Coming from working-class backgrounds, we each carry unique stories that deeply inform our scholarly pursuits and feminist identities. These stories include inspiration from a great-grandmother who was the first woman in her town to enter the workforce, resilience learned from academic mentors who taught us to challenge traditional power dynamics, and encouragement from family members who instilled values of compassion and ambition. Each of us has had some mentors who identified as feminists and other mentors who did not. Each of us has had some mentors who excellently modelled advocacy, empowerment, self-care, and resilience, and other mentors who, while well-intentioned, did not always effectively demonstrate these qualities. These diverse influences have enriched our commitment to feminist research and activism, helping us navigate challenges and fostering empowerment. Feminist mentorship encourages us to challenge traditional power dynamics, embrace resilience, and strive for greater achievements. Many of us owe our academic paths to supportive feminist mentors.

## What is mentorship versus feminist mentorship?

Mentorship, or mentoring, is a construct that is inconsistently defined (Mullen & Klimaitis, 2021; Nuis et al., 2023). However, common elements focus on achievement, emotional and psychological support, career advancement and professional development assistance, role modeling, mutual benefits, personal interaction, and mentors’ having greater experience, power, and achievement (Jacobi, 1991). In a higher education context, discussions about the dynamics of mentoring typically focus on formalized processes (e.g., training, compensation), the developmental relationship, actor functions (e.g., support, role modelling), and achievement (Nuis et al., 2023).

Feminist mentorship incorporates these aspects but aims to disrupt traditional power structures and foster inclusive environments (Benishek et al., 2004). For us students, a critical component of this type of mentorship is the opportunity to learn and grow in environments that prioritize our empowerment. Feminist mentorship emphasizes intersectionality and critical reflection, and addressing gender inequalities (Benishek et al., 2004; Heinrich, 1995). Thus, anyone can practice feminist mentorship by incorporating these elements into their mentoring practices. Feminist mentorship is not tied to the gender of the mentor. This type of mentorship encourages a nurturing and equitable environment that supports mentees’ personal and professional growth (Manongsong & Collins, 2022).

Feminist mentorship benefits women students as they often face disproportionate demands compared to male peers, leading to burnout and increased dropout rates (Bayfield et al., 2020; Dua, 2007; Schmidt & Faber, 2016). Mentors who practice feminist mentoring by actively challenging patriarchal norms and emphasizing equity and diversity can help dismantle barriers that disproportionately impact women, enriching the graduate student experience by improving students’ confidence, self-esteem, and overall satisfaction with their academic program (Dua, 2007; Harris, 2022). For example, feminist mentoring integrates discussions about feminist principles, such as intersectionality and equality, into regular mentorship activities, and provides opportunities for mentees to engage in advocacy, allyship, and research aligned with feminist values. By encouraging graduate students to engage in critical reflection that challenges systemic inequities, considering the experiences of marginalized voices, and fostering an inclusive and supportive community, the unique benefits of feminist mentorship go above and beyond standard mentorship frameworks. These types of discussions, which might not take place in other types of mentorship dynamics, are what have set feminist mentors apart from others in our experiences.

## Mentorship as an introduction to feminism

Feminist mentoring introduces women to feminist principles, fostering an appreciation for feminism's impact on their professional and personal identities (despite, in some circumstances, initial hesitation about adopting feminist ideologies; Simic, 2010). This mentorship, including peer relationships among early career scholars, encourages collaborative learning, shared experiences, and resource-sharing, promoting solidarity and collective growth within an inclusive academic environment. It helps students, especially women and gender minorities, navigate the complexities of academic institutions, leading to positive outcomes for their academic and emotional well-being (Lorenzetti et al., 2019). The support extends across various mentoring dynamics, including faculty–student and peer mentoring relationships. Mentorship is especially critical in facilitating academic success for women and other minoritized groups, empowering them to overcome inherent challenges within academia (Farkas et al., 2019; Nokkala et al., 2022; Schmidt & Faber, 2016).

Feminist mentoring also often provides a deeper understanding of feminism's role in shaping personal and professional identities (Simic, 2010). As some of the authors of this paper experienced, entering graduate school with only a surface-level understanding of feminism can feel intimidating, especially when encountering concepts like radical feminism, hegemonic masculinity, and intersectionality. However, through academic growth, feminist mentoring, and immersion into the history of many feminist scholars and trailblazers, we learned the significance of these concepts and how they are deeply interwoven into the fabric of society. Feminist mentoring offered us a glimpse behind the curtain of surface-level feminism, enabling us to embrace our roles as true feminist activists working to change systemic inequities.

## Role modeling positive behaviors and research practices

As we can attest, graduate students often emulate the behaviors displayed by professors they respect and admire. Some of us have personally observed respected role models experience burnout and become overstretched in their work, which has influenced our perception of what is expected and “normal” in academia, believing we need to mirror these behaviors to be successful. Feminist mentorship models encourage advisors to practice and model self-reflection, self-compassion, and work–life balance, and reflect on the impact their behaviors have on mentees. Accordingly, our mentors who have modeled positive examples of work–life balance—such as regularly taking days off, setting boundaries around checking emails outside work hours, and prioritizing tasks effectively—have provided us with invaluable guidance on maintaining both productivity and personal well-being.

These behaviors help address challenges women face in graduate education, such as overcoming gender bias, balancing academic responsibilities with personal and family obligations, navigating impostor syndrome, and accessing mentorship and networking opportunities (Carter et al., 2013; Morgan et al., 2021; Vaughn et al., 2020; Wall, 2008). Although feminist mentors are not required to be women, women students may also be at a higher risk of feeling isolated or marginalized due to a lack of women faculty mentors (Dua, 2007; Maher et al., 2004; Wall, 2008). Therefore, feminist mentorship should be adopted as a value to provide crucial psychosocial support in male-dominated departments rather than being tied to gender identity. When feminist mentorship is tied to gender identity, it may create an extra burden on women faculty and ignore the responsibility of men faculty in male-dominated departments.

Having a feminist mentor before graduate school can also increase the likelihood that women students will have sufficient learning opportunities to improve their chances of getting into graduate school. Often, women are rejected from graduate school because gender-based barriers create a lack of access to undergraduate-level research engagement activities, such as research assistantships, writing proposals or grants, coauthoring publications, presenting at conferences, or other networking activities (Bruce, 1995; Davidson & Foster-Johnson, 2001; McClure, 2019; Rowell et al., 2020). Even for some authors of this paper, the lack of access to mentorship and undergraduate-level research opportunities made the graduate school application process substantially more challenging.

Feminist mentors can also improve women's chances of completing their programs on time and gaining the experience necessary for job market success. Faculty who adopt a feminist mentorship model are more likely to promote and value collaboration with their students, ensuring they feel valued and recognized for their contributions (e.g., authorship on research papers; Dua, 2007). In our experience, the value of collaborative work with faculty members has been integral to our success, bolstering our curricula vitae (CVs) and preparing us for the job market.

In fostering such support, feminist mentors create nurturing and equitable student environments by advocating diverse research perspectives and methodologies (Arczynski et al., 2018; Humble et al., 2006; Thompson & Walker, 1995). While these practices may align with characteristics of good supervision in general, what distinguishes feminist mentors is their commitment to promoting methodological diversity and encouraging students to critically reflect on dominant research practices. For example, quantitative inquiry is often valued over qualitative inquiry in psychology (Ponterotto & Zárate, 2010; Walsh-Bowers, 2002). Yet, feminist mentors critically question the privileging of quantitative inquiry over qualitative approaches in psychology and actively encourage students to value and use diverse methodologies that best align with their projects’ goals and their lived experiences (Arczynski et al., 2018; Humble et al., 2006; Thompson & Walker, 1995). Furthermore, feminist mentors teach students to incorporate an intersectional lens into their research design and to address social power dynamics by engaging with transparency, collaboration, self-reflection, and contextual awareness tools in the research process (Arczynski et al., 2018).

## Combating impostor syndrome

One major issue that many graduate students face is impostor syndrome, characterized by unwarranted self-doubt about one's competencies despite one's accomplishments and by attributing success to luck rather than ability (Clance & Imes, 1978). Feminist mentors counter impostor syndrome by explicitly acknowledging and validating women’s and marginalized students’ unique academic challenges and accomplishments, normalizing these feelings while helping their students recognize and build confidence in their strengths and abilities. Moreover, by actively addressing gender discrimination, lack of mentorship, and inadequate faculty support, feminist mentors can create inclusive departmental cultures that prioritize gender and race issues, reducing feelings of isolation and preventing attrition (Dua, 2007). Their examples can empower women to recognize their capabilities, boost confidence, and improve assertiveness. By fostering a sense of community and belonging, they help women scholars thrive personally and academically (Dua, 2007).

In our experiences, effective feminist mentoring has played a crucial role in helping us combat impostor syndrome. By fostering a supportive work environment and promoting collaborative efforts, as outlined above, we have recognized our valuable skills and contributions to projects, built confidence, and thrived in our personal and professional development.

## Recommendations for supporting emerging feminist scholars

To conclude this commentary on feminist mentorship, we offer several recommendations for supporting emerging feminist scholars in contemporary academic settings. These recommendations, outlined in Table 2, are for faculty and mentors aiming to better support graduate students. They combine our personal insights as feminist graduate students with academic literature on best mentorship practices. Our recommendations underscore the profound impact of mentorship on growth and empowerment. As a reflection on this commentary, we advocate integrating feminist principles into all mentorship initiatives, as inclusive practices, equity, and diversity should be a part of all graduate student experiences. We encourage academics and mentors to strive toward creating more equitable environments where students feel valued, supported, and empowered to thrive.

**Table 2.** Actionable recommendations for supporting emerging feminist scholars.

**Connect with other feminist labs and spaces while emphasizing and supporting networking and collaboration initiatives outside your lab.** This highlights a commitment to exposing students to diverse perspectives and methodologies. It enhances their access to new resources, generates innovative ideas, fosters professional collaborations, and builds meaningful relationships. **Routinely seeking funding opportunities for students.** Actively and regularly seeking funding opportunities (e.g., scholarships, student grants, travel awards) demonstrates a commitment to students’ success by reducing financial stress. It can also boost their curricula vitae and competitiveness for future work positions. **Provide equitable support and advocacy for students.** This approach highlights a commitment to equitable support by ensuring that all students, regardless of their background, have access to the resources and guidance they need to succeed. This demonstrates a commitment to addressing systemic inequities, fostering trust, and providing opportunities that might otherwise be inaccessible. **Engage with a broad, diverse range of content, theoretical frameworks, research materials, and interdisciplinary perspectives to share with students.** This approach demonstrates a commitment to preparing students to address complex, intersectional issues in their research and future careers. Promoting engagement with frameworks from other disciplines also fosters creativity and versatility in student work. **Tailor mentorship approaches to individual students’ needs, acknowledging that some students require different supervision levels than others**. Tailoring mentorship reflects a commitment to respecting students’ unique needs and goals by acknowledging that different levels of guidance are necessary for growth, especially for mentees at different levels of their careers. **Promote community and peer support within the lab.** This demonstrates a commitment to combating isolation and creating an environment that aligns with feminist values. A strong lab community helps students navigate challenges, celebrate successes, and develop a sense of belonging, peer support, and collective achievement. **Challenge traditional academic hierarchies and power structures to promote empowerment and individual growth.** This highlights a commitment to equity and inclusivity by creating spaces where students are treated as equals and feel empowered to voice their ideas. Challenging traditional hierarchies supports open communication, personal growth, and confidence to challenge norms. **Address gender inequity and promote inclusion and community-based activism (e.g., advocating equal opportunity policies, creating inclusive learning environments, and challenging systemic barriers).** Addressing systemic barriers and promoting equity demonstrates a commitment to fostering inclusion and empowering marginalized students. **Support undergraduate and graduate students in personal and professional growth.** This approach highlights a commitment to holistic mentorship by recognizing the connection between personal and professional development. Supporting students in both areas helps them build resilience, self-awareness, and the skills necessary for future success. **Model reflexivity, personal reflection, self-compassion, and work–life balance.** These behaviors highlight a commitment to challenging academic norms that glorify overwork while demonstrating that success can be achieved without sacrificing well-being, prioritizing mental health and adopting sustainable work habits. **Model adaptability, empathy, and concern for others in academic spaces.** This practice reflects a commitment to creating inclusive and supportive academic environments where students feel valued and understood and develop essential skills for leadership and collaboration in their future endeavors. **Recognize the importance of early mentorship (e.g., support during undergraduate programs to increase success in graduate school applications, especially for students from underrepresented groups).** This approach provides students with the tools, guidance, and confidence to navigate academic pathways and pursue advanced opportunities.
